# Epidemiological and clinical aspects of paediatric HIV infections in Setif (Algeria)

**DOI:** 10.1186/1471-2334-14-S2-P14

**Published:** 2014-05-23

**Authors:** A Ouyahia, M Rais, A Gasmi, S Mechakra, A Lacheheb

**Affiliations:** 1 Ferhat Abbes University, faculty of medicine, Setif, Algeria

## Background

Algeria has a low HIV prevalence and data on the risk factors and clinical presentation of HIV-infected children are lacking.

## Objective

To describe the epidemiological, clinical characteristics and outcome of HIV-infected children in Setif.

## Methods

A retrospective review was undertaken of the medical records of all patients admitted to the HIV unit of the teaching hospital of Setif which serves a population from a wide geographic catchment area, between January 2002 and November 2013.

## Results

Of 286HIV-infected patients, 12 were children (4.1%), 8 (66.7%) of whom were male. Ages at diagnosis ranged from 6 months to 12 years (median 5).

Of the 10 (83.3%) children who acquired the infection by vertical transmission, median age at diagnosis was 3 years. Only one woman was offered PMTCT interventions with HAART started lately at 38 weeks of pregnancy, oral AZT and replacement feeding to neonate.

All neonates were delivered by spontaneous vaginal delivery, 9 of them were breastfed throughout infancy.

Children commonly presented with prolonged fever (25%), recurrent cough (n=33.3 %), failure to thrive (n=41.66 %) and recurrent diarrhoea (n=8.3%). One case had tuberculosis associated to herpes zoster, 2 cases of moluscum contagiosum, one case of anal condyloma and one case of visceral leishmaniosis. All cases received highly active antiretroviral. Of those 91.6% were compliant with treatment and had a sustained virologic response below the detectable levels. One child died at age of 6 months.

## Conclusion

The majority of HIV-infected children presenting to our hospital acquired HIV through vertical transmission. A strong prima facie case for the introduction of mandatory HIV testing of pregnant women must be made because the price paid by HIV-infected newborns for their mothers’ failure to undergo testing and treatment is very high.

